# Comparison of exenatide alone or combined with metformin versus metformin in the treatment of polycystic ovaries: a systematic review and meta-analysis

**DOI:** 10.1186/s12902-023-01497-x

**Published:** 2023-11-16

**Authors:** Yan Hu, Xiangxin Song, Shaila Hamiti, Yanyong Ma, Mainu Yusufu, Xing Wang, Kaidi Zhang, Yanying Guo

**Affiliations:** 1 Department of Endocrinology, People’s Hospital of Xinjiang Uygur Autonomous Region, Xinjiang Clinical Research Center for Diabetes, Urumqi, Xinjiang, China; 2 Graduate School, Xinjiang Medical University, Urumqi, Xinjiang, China

**Keywords:** Exenatide, Pregnancy rate, Gonadal steroid hormones, Weight loss, Insulin resistance

## Abstract

**Background:**

Polycystic ovary syndrome (PCOS) is the most common cause of anovulatory infertility in women of childbearing age. Randomized controlled trials (RCTs) have reported that exenatide and metformin are effective in the treatment of PCOS. In this meta-analysis, we aimed to compare the effectiveness and safety of exenatide alone or in combination with metformin versus metformin in patients suffering from PCOS.

**Methods:**

RCTs of exenatide therapy were identified through a search of electronic databases in November 2022 and updated in October 2023. Eligible studies were identified independently by the reviewers. Outcomes were analysed with Revman 5.4.

**Results:**

Nine RCTs among 214 studies on 1059 women with PCOS were included in the analysis, and among the nine RCTs, eight studies compared exenatide with metformin. Our meta-analysis demonstrated that exenatide was more effective than metformin in terms of pregnancy rate (RR 1.85 [95% CI 1.19,2.86] *P* = 0.006), sex hormone-binding globulin (SHBG) (MD 5 [95% CI 3.82,6.18] *P* < 0.001), and follicle-stimulating hormone (FSH) (MD 0.82 [95% 0.41,1.24] *P* < 0.001). The reductions in total testosterone (TT) (SMD -0.43 [95% CI -0.84, -0.03] *P* = 0.04) was more significant after treatment with exenatide than after treatment with metformin. In terms of safety, exenatide had a lower diarrhea rate (RR 0.11 [95% CI 0.01, 0.84]) than metformin. In the other three studies, exenatide plus metformin was compared with metformin. Exenatide combined with metformin was more effective in improving SHBG (MD 10.38[95%CI 6.7,14.06] *P* < 0.001), Matsuda index (MD 0.21[95%CI 0.05,0.37]) and reducing free androgen index (FAI) (MD -3.34 [-4.84, -1.83] *P* < 0.001), Weight (MD -2.32 [95%CI -3.89, -0.66]) and WC (MD-5.61[95%CI -8.4, -2.82] *P* < 0.001). The incidence of side effects between exenatide plus metformin and metformin was not statistically significant.

**Conclusions:**

Exenatide alone or in combination with metformin is more effective than metformin for women with PCOS. Considering the evidence on effectiveness and safety, exenatide alone or in combination with metformin may be a better treatment approach than metformin for women with PCOS.

**Trial registration:**

INPLASY https://inplasy.com/inplasy-protocols/ ID: 10.37766/inplasy2022.11.0055.

**Supplementary Information:**

The online version contains supplementary material available at 10.1186/s12902-023-01497-x.

## Introduction

Polycystic ovary syndrome (PCOS) is the most common endocrine disorder among women of childbearing age and is associated with high androgen levels, oligomenorrhea or amenorrhea, anovulation, and polycystic morphology of the ovaries, according to the Rotterdam criteria [1]. Although insulin resistance and obesity are not necessary criteria for diagnosing PCOS, they are important pathophysiological alterations in PCOS patients [2, 3]. Insulin resistance and obesity alone or in combination with sex hormone disorder [4, 5] may gradually give rise to reproductive and metabolic abnormalities [6, 7], cardiovascular disease [8], diabetes mellitus [9], and non-alcoholic fatty liver [10].

Clinical studies have demonstrated that metformin improves anovulation, menstrual disturbances, and hyperandrogenism in patients with PCOS [11, 12]. Metformin has also been prescribed for obese women with PCOS who suffer from metabolic irregularity on basis of the guidelines for the assessment and management of PCOS [13]. However, metformin has a slow onset of action, and it is difficult to achieve satisfactory weight control with metformin in patients with PCOS.

Exenatide, which belongs to the glucagon-like peptide-1 (GLP-1) receptor agonist family, has been used to treat diabetes and obesity chiefly owing to its prominent effects on insulin resistance, weight loss, and metabolic disorders [14, 15]. Some clinical randomized controlled trials (RCTs) have demonstrated that exenatide also has beneficial effects on pregnancy rate [16, 17], the menstrual frequency ratio (MFR) [18], insulin resistance (IR) [19], and weight reduction in women who are suffering from PCOS [16, 20]. Whether exenatide is the preferred treatment option for patients with PCOS still needs to be confirmed by large-scale clinical studies and adequate evidence-based data. When choosing a drug regimen, it is necessary to consider efficacy and safety. The most common adverse reactions associated with exenatide treatment are gastrointestinal reactions [21]. Other side effects include sclerosis at the injection site, headache, and allergic reactions [22]. Therefore, in this meta-analysis, we aimed to compare effectiveness and safety of exenatide or exenatide plus metformin versus metformin for women suffering from PCOS.

## Methods

### Search strategy

Original studies published in English for this meta-analysis were sought from electronic databases (PubMed, Embase, Web of Science, Cochrane library, the Web of Science, Scopus, and Google Scholar). We searched for RCTs on humans in databases from their establishment date to October 2023 by utilizing a strategy combining MeSH words with free words. The utilized terms involved "Polycystic Ovary Syndrome" [MeSH], Ovary Syndrome, Polycystic [Title/Abstract], Syndrome, Polycystic Ovary [Title/Abstract], "Exenatide"[MeSH], Exendin-4 [Title/Abstract], Peptide, Ex4 [Title/Abstract], randomized controlled trial [Publication Type], and randomized [Title/Abstract]. The search history on PubMed is presented in Table S1.

### Inclusion and exclusion criteria

All searched articles were independently screened by 2 authors according to the inclusion criteria, which were as follows: 1) participants: females diagnosed with PCOS on the basis of the Rotterdam criteria; 2) intervention(s): exenatide alone or plus metformin in the treatment of PCOS; 3) comparison(s): exenatide versus metformin; 4) outcomes: effectiveness estimated by pregnancy rate, sex hormone levels, change in body weight, and metabolic disorders and safety assessed by the incidence of side effects; 5) study type: human-based RCTs. The exclusion criteria were as follows: one-arm study with no control group, placebo-controlled trials, and studies with no results.

### Data extraction

Two authors (XS and SH) selected articles independently based on the inclusion and exclusion criteria and then reviewed the full texts to extract data from the eligible articles. In the case of disputes, a discussion meeting was organized by a third author (YM). Necessary information for outcomes included pregnancy rate, MFR, total testosterone (TT), follicle-stimulating hormone (FSH), luteinizing hormone (LH), dehydroepiandrosterone sulfate (DHEAS), sex hormone-binding globulin (SHBG), the free androgen index (FAI), homeostasis model assessment-insulin resistance (HOMA-IR), Matsuda index, fasting insulin (FINS), fasting blood glucose (FBG), weight, body mass index (BMI), waist circumference (WC), the waist-hip ratio (WHR), and side effects (diarrhea, constipation, nausea, vomiting, stomach pain, headache, and fatigue). Information on the trials included basic information (authors, publication year, title, criteria for PCOS, number of attendees, mean age, and total sample sizes), interventions (drugs, dose, and duration), and study design. The MFR was calculated using the ratio of expected menses to observation weeks. Matsuda index is an indicator of insulin sensitivity calculated from the glucose tolerance test.

### Risk of bias assessment and quality assessment

The risk of bias for each RCT was evaluated by using the Cochrane Collaboration’s risk of bias assessment, which includes the aspects of sequence generation, allocation concealment, blinding, incomplete outcome data, selective outcome reporting and other bias. Studies were characterized as “low risk”, “unclear risk”, and “high risk”. Quality assessment of the RCTs was performed with the Grading of Recommendations and Evaluation (GRADE) system. Quality was defined as “high”, “moderate”, “low”, or “very low” according to the study design, risk of bias, indirectness, inconsistency, imprecision, and publication bias [23].

### Statistical analysis

Meta-analysis plus forest plots were applied by utilizing Rev Man 5.4. Continuous variables were presented as the standardized mean difference (SMD) or mean difference (MD) with a 95% confidence interval (CI) on basis of the agreement of research units. For dichotomous data, the risk ratio (RR) with 95% CI for every original study was gathered for meta-analysis utilizing the Mantel/Haenszel model. Heterogeneity was appraised through the Chi-squared (X^2^) test and presented as I-squared (I^2^) values. I^2^ < 50% and *P* > 0.1 indicate the existence of little heterogeneity among original studies. Analyses were employed by utilizing fixed-effects models. I^2^ ≥ 50% indicates high heterogeneity, and subgroup analysis or sensitivity analysis was performed. If no reason for the heterogeneity could be determined and the heterogeneity was within the relevant limits, a random-effects model could be utilized. *P* values < 0.05 were recognized to indicate significant differences.

## Results

### Study identification

**Fig. 1:**
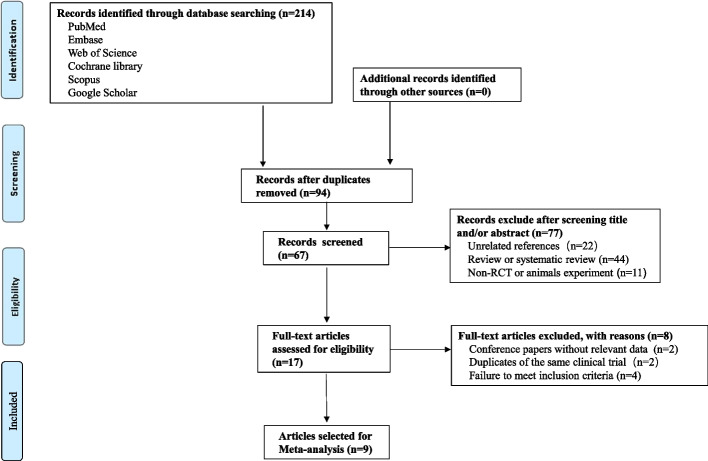
Diagram of RCTs selection

All 214 articles were obtained after initial examination of the electronic databases. Nine RCTs were ultimately included after removing duplicates, screening the titles and abstracts, and performing the whole study assessment [21, 24–30]. The procedures used to search for and identify RCTs are shown in Fig. 1. Details of excluded articles in the supplementary Excel sheet.

### Detailed information on the selected trials

A total of 1149 women with PCOS were included in the 9 RCTs. One of the 9 trials had an intervention time of 24 weeks [31], while the remaining trials had an intervention time of 12 weeks. The total sample sizes ranged from 40 to 176. The dose of exenatide ranged from 10 µg/day (5 µg bid) to 20 µg/day (10 µg bid), while the dose of metformin ranged from 1000 mg/day (500 mg bid) to 3000 mg/day (1000 mg tid). Detailed information on the selected trials is reported in Table 1.

**Table 1:** Characteristics of the included studies

Author (year)	Study design	PCOS criteria	Age (years) (T/C)	Regimen		Total sample sizes	Duration (weeks)
				Experimental group	Control group		
Comparison between exenatide versus metformin							
Elkind-Hirsch. K et al.,2008 [31]	Randomized, clinical, prospective, outpatient trial	Rotterdam 2003 criteria	(28.2 ± 1.1)/(27.7 ± 1.3)	Exenatide 10ug bid	Metformin 1000 mg bid	40	24Weeks
Li. R.Y et al., 2020 [17]	Randomized, clinical, prospective trial	NA	NA	Exenatide	Metformin	147	12Weeks
Li. R et al.,2022 [16]	Randomized, clinical, prospective trial	Rotterdam criteria	(28.22 ± 3.89)/(27.78 ± 3.81)	Exenatide 5 μg bid	Metformin 1000 mg bid	160	12Weeks
Liu. X et al.,2017 [18]	Randomized, clinical, prospective trial	Rotterdam criteria	(27.93 ± 2.7)/(27.69 ± 3.80)	Exenatide 10 μg bid	Metformin 1000 mg bid	176	12Weeks
Tao. T et al.,2021 [32]	Randomized, clinical, parallel-group controlled trial	Rotterdam 2003 criteria	NA	Exenatide 10 μg bid	Metformin 1000 mg tid	100	12Weeks
Wang. J et al., 2017 [33]	Randomized, clinical, single-center, controlled, single-blind trial	Rotterdam criteria	(25.92 ± 6.75)/(25.67 ± 7.33)	Exenatide 10 μg bid	Metformin 500 mg bid	78	12Weeks
Zheng. S et al.,2017 [10]	Randomized, clinical, prospective trial	Rotterdam 2003 criteria	(27.20 ± 3.1)/(27.7 ± 2.7)	Exenatide 10 μg bid	Metformin 1000 mg bid	176	12Weeks
Zheng. S et al.,2019 [20]	Randomized, clinical, prospective trial	Rotterdam criteria	(27.2 ± 1.76)/(27.7 ± 1.64)	Exenatide 10 μg bid	Metformin 1000 mg bid	82	12Weeks
Comparison between exenatide + metformin and metformin alone							
Elkind-Hirsch. K et al.,2008 [31]	Randomized, clinical, prospective, outpatient trial	Rotterdam 2003 criteria	(32.1 ± 0.7)/(27.7 ± 1.3)	Exenatide 10 μg bid + Metformin 1000 mg bid	Metformin 1000 mg Bid	40	24eeks
Ma. R. L et al.,2021 [21]	Randomized, clinical, prospective, outpatient trial	Rotterdam criteria	(30.10 ± 4.52)/(28.17 ± 4.40)	exenatide 2 mg qw + Metformin 500 mg tid	Metformin 500 mg tid	50	12Weeks
Tao. T et al.,2021 [32]	Randomized, clinical, parallel-group controlled trial	Rotterdam 2003 criteria	NA	Exenatide 10 μg bid + Metformin 1000 mg tid	Metformin 1000 mg tid	100	12Weeks

## Quality assessment

**Fig. 2:**
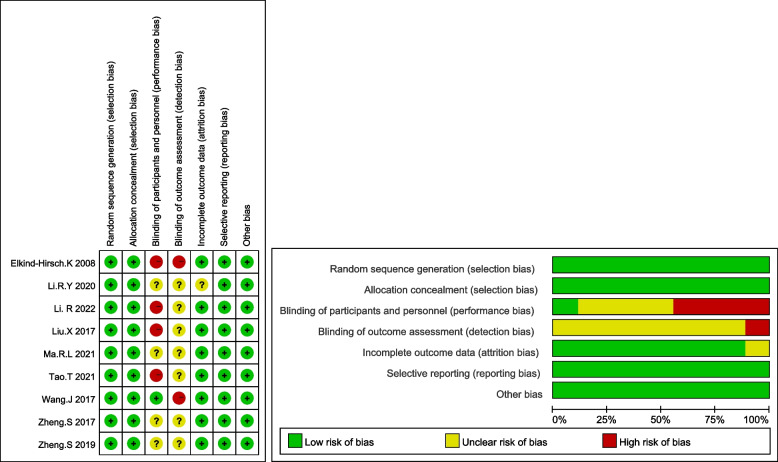
The risk of bias assessment of RCTs

The risk of bias estimation of the RCTs is displayed in Fig. 2, and the details can be found in Table S2. All 9 selected trials had a clear description of random sequence generation and allocation concealment. Four trials clearly stated that interventions were not blinded to participants and personnel [16, 18, 31, 32], one trial was blinded to participants and personnel [33], and the other four trials did not report details of the blinding method [10, 17, 20, 34]. One trial clearly stated that the blinding method was not applied to outcome assessors [33], while the remaining eight trials did not report whether blinding was applied to outcome assessors. The risk of incomplete outcome data in one study was uncertain [17], while in the other eight studies, it was low. The risk of reporting bias and other bias was low in all trials. The quality of the included studies was assessed by using GRADE and is presented in Table 2. The included studies were of moderate to low quality, and the main negative points were risk of bias and imprecision.

**Table 2:** Quality assessment of the studies

Author (year)	Type of study	Factor of downgrade					Factor of escalation	Level of evidence
		Risk of bias	Inconsistency	Indirectness	Imprecision	Publication bias		
Elkind-Hirsch. K et al., 2008 [31]	RCT	-1	0	0	-1	0	0	Low
Li. R.Y et at 2020 [17]	RCT	-1	0	0	0	0	0	Medium
Li. R et al.,2022 [16]	RCT	-1	0	0	0	0	0	Medium
Liu. X et al.,2017 [18]	RCT	-1	0	0	0	0	0	Medium
Tao. T et al.,2021 [32]	RCT	-1	0	0	0	0	0	Medium
Wang. J et al., 2017 [33]	RCT	-1	0	0	-1	0	0	Low
Zheng. S et al.,2017 [10]	RCT	0	0	0	0	-1	0	Medium
Zheng. S et al.,2019 [20]	RCT	0	0	0	-1	0	0	Medium
Ma. R. L et al.,2021 [21]	RCT	-1	0	0	0	0	0	Medium

### Comparison between exenatide and metformin

#### Comparison of the effectiveness between exenatide and metformin

**Fig. 3:**
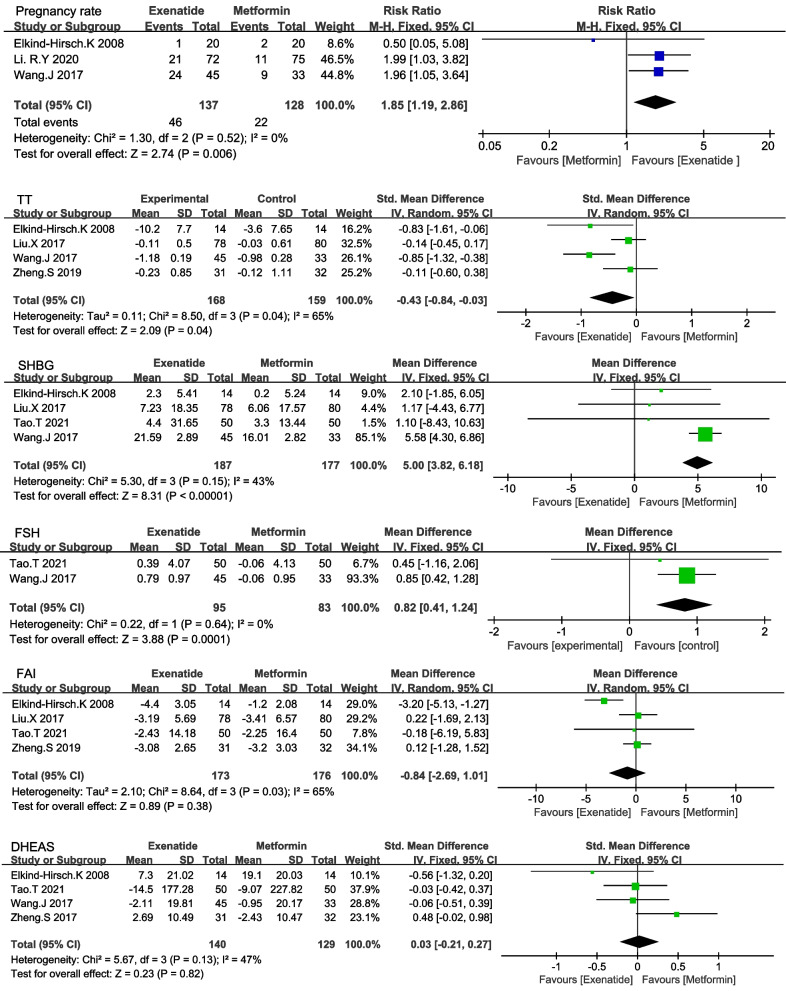
Effects on pregnancy and sex hormone levels between exenatide and metformin

Three trials reported the outcome of pregnancy rate which included 265 women with PCOS. There was no heterogeneity between trials (I^2^ = 0%, *P* = 0.52), and the fixed-effects model was used for examination. Exenatide had a higher pregnancy rate (RR 1.85 [95% CI 1.19,2.86] *P* = 0.006) than metformin (Fig. 3) (Table 3).

**Table 3:** The meta-analysis of pregnancy rate

No. of studies included	No. of cases (T/C)	Heterogeneity test		Meta-analysis results	
		P	I^2^	RR (95% CI)	*P* value
3	137/128	0.52	0%	1.85(1.19,2.86)	0.006

Four studies including 327 women reported TT, and the heterogeneity among studies was significant (I^2^ = 65%, *P* = 0.04). The random effects model of the meta-analysis indicated that exenatide is superior to metformin in TT (SMD -0.43 [95% CI -0.84, -0.03] *P* = 0.04) (Fig. 3) (Table 4). Sensitivity analysis was conducted due to the significant heterogeneity observed among the four included studies (I^2^ = 65%, *P* = 0.04). This analysis indicated that the study "Elkind-Hirsch K 2008" greatly contributed to the observed heterogeneity. After excluding this study, heterogeneity testing was insignificant (I^2^ = 0%, *P* = 0.51). The heterogeneity in the excluded study could possibly be attributed to its small sample size and the distinct population compared to the other three studies. The Meta-analysis results remained consistent after sensitivity analysis, suggesting the stability of the findings. (MD = -0.16 [95% CI (-0.26, -0.07)] *P* < 0.001) (Fig S3).

**Table 4:** The meta-analysis of effectiveness

Outcomes	Comparability of baseline	No. of studies included	No. of cases (T/C)	Heterogeneity test		Meta-analysis results	
				P	I^2^	MD/SMD (95% CI)	*P* value
Comparison between exenatide versus metformin							
TT	comparable	4	168/159	0.04	65%	-0.43(-0.84, -0.03)	0.04
SHBG	comparable	4	187/177	0.15	43%	5(3.82,6.18)	< 0.001
FSH	comparable	2	95/83	0.64	0%	0.82(0.41,1.24)	< 0.001
FAI	comparable	4	173/176	0.03	65%	-0.84(-2.69,1.01)	0.38
DHEAS	comparable	4	140/129	0.13	47%	0.03(-0.21,0.27)	0.82
MFR	comparable	2	92/94	< 0.001	97%	0.16(-0.01,0.34)	0.07
HOMA-IR	comparable	5	243/234	0.16	39%	-0.68(-0.84, -0.53)	< 0.001
FINS	comparable	4	229/220	0.82	0%	-0.61(-0.8, -0.42)	< 0.001
FBG	comparable	4	229/220	< 0.001	87%	-0.1(-0.29,0.09)	0.30
Weight	comparable	5	248/251	1	0%	-1.69(-2.49, -0.89)	< 0.001
BMI	comparable	5	248/251	0.03	63%	-1.13(-1.82, -0.43)	0.002
WC	comparable	4	198/201	0.4	0%	-2.49(-3.29, -1.7)	< 0.001
WHR	comparable	3	184/187	0.57	0%	-0.02(-0.03, -0.01)	< 0.001
Comparison between exenatide + metformin and metformin alone							
SHBG	comparable	2	64/64	0.41	0%	10.38(6.7,14.06)	< 0.001
FAI	comparable	2	64/64	0.35	0%	-3.34(-4.84, -1.83)	< 0.001
Matsuda index	comparable	2	33/35	0.88	0%	0.21(0.05,0.37)	0.01
Weight	comparable	3	83/85	0.3	17%	-2.32(-3.89, -0.66)	0.006
WC	comparable	2	33/35	0.28	14%	-5.61(-8.4, -2.82)	< 0.001

*MFR* Menstrual frequency ratio, *TT* Total testosterone, *FSH* Follicle stimulating hormone, *SHBG* Sex hormone binding globulin, *LH* Luteinizing hormone, *DHEAS* Dehydroepiandrosterone sulphate, *FAI* Free androgen index, *HOMA-IR* Homeostasis model assessment-insulin resistance, *FINS* Fasting insulin, *FBG* Fasting blood glucose, *BMI* Body mass index, *WC* Waist circumference, *WHR* Waist-hip ratio

The pooled outcomes for SHBG in four studies and FSH in two studies were reported between the two groups. The heterogeneity between trials was low, and a fixed-effects model was used for analysis. Exenatide was more beneficial than metformin in terms of SHBG (MD 5 [95% CI 3.82,6.18] and FSH (MD 0.82 [95% 0.41,1.24] P < 0.001) (Fig. 3) (Table 4).

The exenatide and metformin groups exhibited similar effects on FAI (SMD -0.84 [95% CI -2.69,1.01] *P* = 0.38) and DHEAS (SMD 0.03 [95% CI -0.21,0.27] *P* = 0.82) (Fig. 3) (Table 4). Due to the significant heterogeneity among the four included studies for FAI (I^2^ = 65%, *P* = 0.03), a sensitivity analysis was performed. This analysis identified the study "Elkind-Hirsch K 2008" as a major contributor to the observed heterogeneity. Upon excluding this study, the heterogeneity testing was insignificant (I^2^ = 0%, *P* = 0.99). The observed heterogeneity may be due to its small sample size and unique population compared to the other three studies. The Meta-analysis results remained consistent after conducting the sensitivity analysis, indicating the robustness of the findings (MD = 0.14 [95% CI (-0.97, 1.26)] *P* = 0.8) (Fig S3).

The MFR was reported in two studies comparing exenatide and metformin in 256 patients with PCOS. Exenatide showed greater effectiveness than metformin in improving MFR, however, there was significant heterogeneity among the two studies included (I^2^ > 97%) (Table 4).

Meta-analysis demonstrated a greater reduction in HOMA-IR, FINS, weight, BMI, WC and WHR in the exenatide group than in the metformin group. The difference in FBG between the two groups was insignificant (Fig S1, Fig S2). Owing to the heterogeneity among the four studies included in the meta-analysis of FBG, we conducted subgroup analyses based on the dosages of exenatide and metformin (Fig S4). Similarly, the five studies included for BMI also exhibited heterogeneity, hence, they were subjected to subgroup analyses as well (Fig S5).

#### Comparison of the safety between exenatide and metformin

**Fig. 4:**
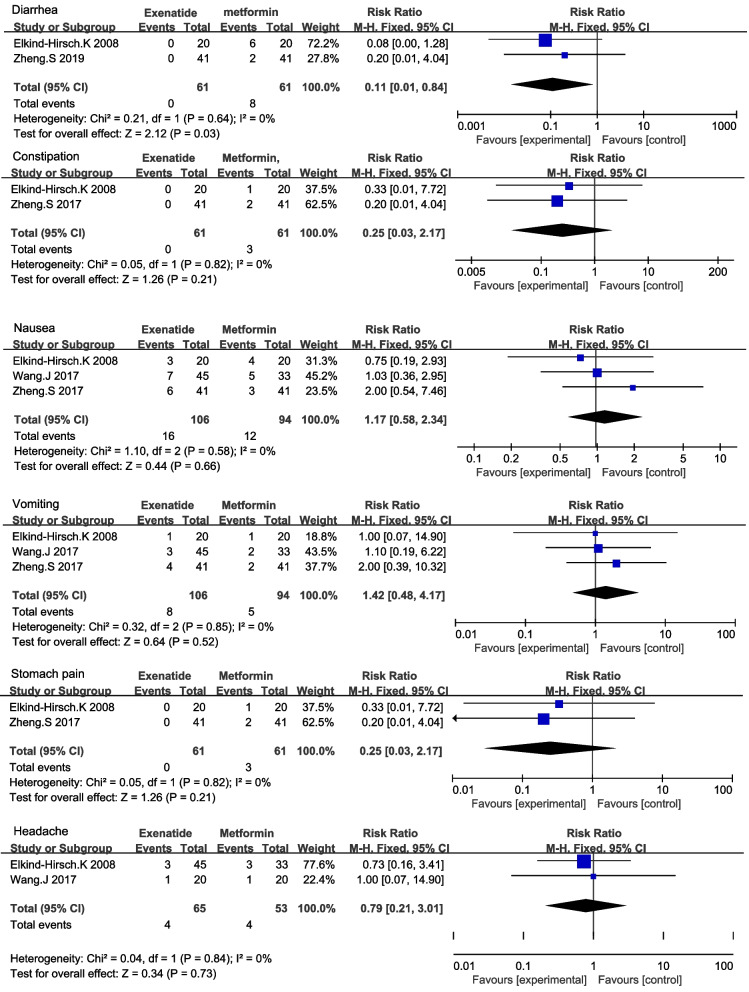
Side effect between exenatide and metformin

Three trials including 122 women with PCOS reported the outcome of diarrhea. There was no heterogeneity among trials (I^2^ = 0%, *P* = 0.64), and the fixed-effects model was used for analysis. Exenatide had a lower diarrhea rate than metformin (RR 0.11 [95% CI 0.01, 0.84] *P* = 0.03) (Fig. 4) (Table 5). The incidence of constipation, nausea, vomiting, stomach pain, and headache in the two groups was not significantly different (Fig. 4) (Table 5).

**Table 5:** The meta-analysis of safety

Type of adverse events	No. of studies included	No. of cases (T/C)	Heterogeneity test		Meta-analysis results	
			P	I^2^	RR (95% CI)	*P* value
Comparison between exenatide versus metformin						
Diarrhea	2	61/61	0.64	0%	0.11(0.01,0.84)	0.03
Nausea	3	106/94	0.58	0%	1.17(0.58,2.34)	0.66
Vomiting	3	106/94	0.85	0%	1.42(0.48,4.17)	0.52
Stomach pain	2	61/61	0.82	0%	0.25(0.03,2.17)	0.21
Constipation	2	61/61	0.82	0%	0.25(0.03,2.17)	0.21
Headache	2	65/53	0.84	0%	0.79(0.21,3.01)	0.73
Fatigue	2	61/61	0.14	55%	0.99(0.04,23.27)	1
Comparison between exenatide + metformin and metformin alone						
Diarrhea	2	45/45	0.27	19%	0.65(0.35,1.21)	0.17
Nausea	2	45/45	0.24	29%	1.43(0.83,2.46)	0.2
Vomiting	2	45/45	0.45	0%	1(0.27,3.76)	1
Stomach pain	2	45/45	0.82	0%	0.25(0.03,2.16)	0.21
constipation	2	45/45	0.7	0%	1.5(0.26,8.56)	0.65
Headache	2	45/45	0.37	0%	1(0.18,5.54)	1
Fatigue	2	45/45	0.45	0%	1(0.27,3.76)	1

### Comparison between exenatide plus metformin and metformin

#### Comparison of the effectiveness between exenatide plus metformin and metformin

**Fig. 5:**
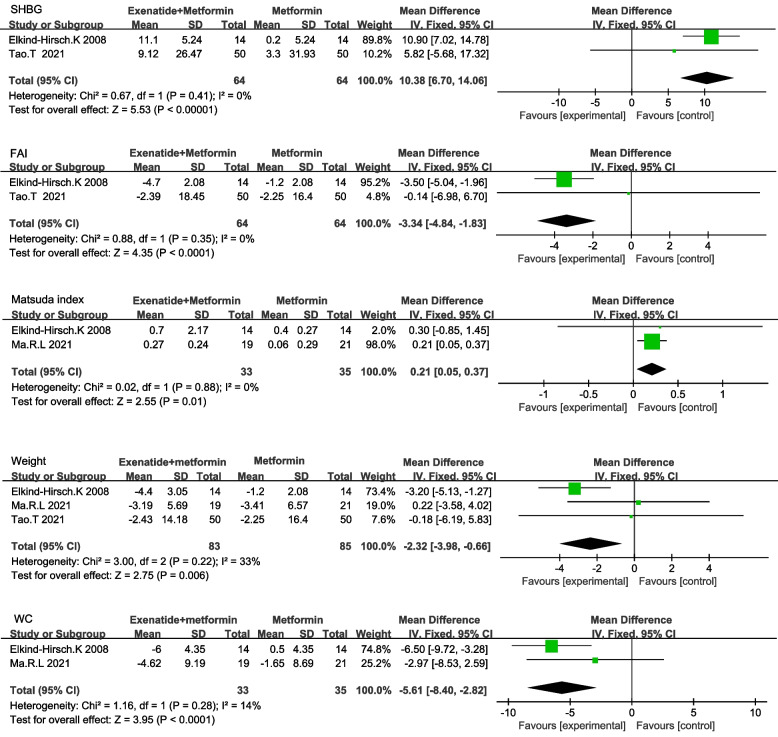
The effectiveness between exenatide plus metformin and metformin

Pooled outcomes of SHBG and FAI in three RCTs were reported in two groups. The heterogeneity among trials was low, fixed-effects model was utilized for analysis. Exenatide plus metformin was more effective in terms of SHBG (MD 10.38[95%CI 6.7,14.06] *P* < 0.001), and FAI (MD -3.34 [-4.84, -1.83] *P* < 0.001) (Fig. 5) (Table 4).

68 women with Matsuda index were reported in two studies, in which the heterogeneity was low. The fixed-effects model of meta-analysis revealed that exenatide plus metformin was superior to metformin in Matsuda index (MD 0.21[95%CI 0.05,0.37] *P* = 0.01) (Fig. 5) (Table 4).

Weight was reported in 3 RCTs involving 168 patients. Meta-analysis utilizing the fixed effects model demonstrated greater reduction in weight (MD -2.32 [95%CI -3.89, -0.66] *P* = 0.006) in the group of exenatide plus metformin (Fig. 5) (Table 4).

WC was reported in 2 studies in the comparison between exenatide plus metformin and metformin containing 68 patients with PCOS. The fixed-effects model was applied for meta-analysis. Results revealed that exenatide was more effective in reducing WC (MD-5.61[95%CI -8.4, -2.82] *P* < 0.001) (Fig. 5) (Table 4).

#### Comparison of the effectiveness between exenatide plus metformin and metformin

**Fig. 6:**
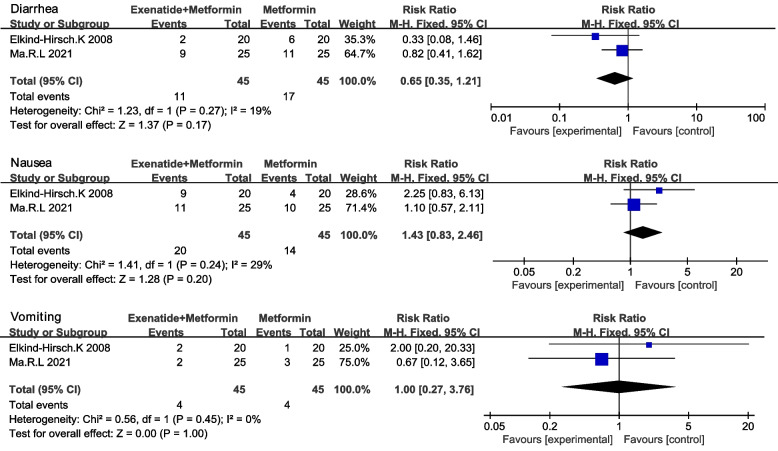
The safety between exenatide plus metformin and metformin

The incidence of diarrhea, nausea, vomiting, stomach pain, constipation, headache, and fatigue between two group was not statistically significant (Fig. 6) (Table 5).

## Discussion

PCOS is commonly associated with infertility, menstrual cycle disorders and abnormal sex hormone levels [5]. In addition, obesity and insulin resistance are frequently seen in women with PCOS and aggravate the adverse features of the syndrome [2]. Exenatide, a GLP-1 receptor agonist, was originally used for glycaemic control in diabetes and was later used for the treatment of obesity [35]. Recently, clinical studies have proven that exenatide also has various beneficial effects on menstrual disorders, anovulation, and androgen excess in patients with PCOS [18, 31, 33]. RCTs reported that exenatide alone or combined with metformin was superior to metformin for weight loss in PCOS patients [16, 18]. However, there is a lack of evidence-based recommendations related to the effects of exenatide on PCOS. This meta-analysis showing that exenatide or exenatide plus metformin is more effective than metformin. Exenatide might affect the human reproductive system through different mechanisms (Fig S6).

The ability of exenatide to improve sex hormone disorders could be one of the reasons for this effect. It is well known that PCOS is the most common cause of anovulatory infertility [36]. Initial data indicate that sex hormone disorders and derangement of early follicle development may be partly responsible for anovulation and infertility in PCOS patients [37]. High levels of circulating androgens can prevent the development of dominant follicles [37]. In addition, adipose tissue aromatizes androgens into oestrogens and affects gonadotropin production by inhibiting the hypothalamic-pituitary-ovarian axis through negative feedback [38]. Reduced hepatic production of SHBG in PCOS patients further limits the bioavailability of peripheral androgens [32]. Therefore, the increase in FSH and SHBG along with the decrease in testosterone results in better regularity of menses and fertility potential. Exenatide was superior to metformin in reducing serum testosterone concentrations and improving FSH and SHBG levels in women with PCOS. Finally, the fertility utility of the exenatide group was better than that of the metformin group, and the pregnancy rate of the exenatide group was significantly higher than that of the metformin group.

The second mechanism for the improvement of reproductive function is based on the improvement of IR and hyperinsulinaemia. PCOS is associated with a high prevalence of IR, hyperinsulinaemia, and high androgen levels [39], particularly in those with abdominal obesity [3, 40, 41]. In addition, PCOS patients with IR are most at risk of infertility, type 2 diabetes (T2DM) and cardiovascular disease [28]. The continuous increase in insulin levels stimulates the ovaries to secrete excessive androgens [42] and decrease hepatic SHBG, ultimately aggravating abnormalities in the hypothalamus-pituitary-ovarian axis, ovulation disorders and infertility [43]. HOMA-IR calculated from FPG and FINS and the Matsuda index derived from fasting and postprandial insulin assays are both indicators of IR [44]. Both metformin and exenatide can reduce IR [21, 45]. Exenatide improves IR partly associated with weight loss and improved glucose metabolism in patients with PCOS [15]. At the same time, exenatide improves IR by inhibiting appetite and glucagon secretion, which delays gastric emptying [28]. The pooled outcomes for HOMA-IR in 5 RCTs were consistent with those of Han Y et al. [40], who reported that HOMA-IR showed better improvements with GLP-1 receptor agonists than with metformin in patients with polycystic ovary syndrome. Based on 4 studies, our meta-analysis showed a greater reduction in FINS in the exenatide group than in the metformin group.

The third mechanism for the improvement of reproductive function involves weight reduction [18], and this effect is mainly achieved through the reduction of the total fat rate, the improvement of IR, and the downregulation of inflammatory factors accompanied by weight loss [31]. The prevalence of obesity or overweight in patients with PCOS is nearly 50% [46]. IR, abdominal fat deposition, and increased androgen secretion in PCOS are exacerbated when obesity is present [47]. Studies have shown that obesity leads to lower implantation and clinical pregnancy rates and higher miscarriage rates [48, 49]. According to international guidelines, women with obesity are recommended to lose weight before spontaneous conception or conception through in vitro fertilization (IVF) [50]. As a short-acting GLP-1 agonist, exenatide has demonstrated positive effects on weight reduction, BMI, WC, and body fat content in overweight/obese PCOS patients [28, 32]. The mechanism by which exenatide mediates weight loss is primarily a reduction in food intake due to direct hypothalamic effects. Exenatide can also delay gastric emptying through central action mediated by the autonomic nervous system. The present meta-analysis confirmed that exenatide showed greater reductions in weight, BMI, WC, and the WHR than metformin.

### Strengths and limitations

The present study has several strengths. Our study compared the efficacy and safety between exenatide alone or plus metformin and metformin in patients with PCOS from several perspectives. We not only focused on exenatide, but also summarized the effectiveness of exenatide plus metformin in PCOS patients, which previous meta-studies on GLP-1 receptor agonists have not done.

Our research also has some limitations. To date, the clinical randomized controlled trials of exenatide in the treatment of PCOS have been short-term trials of 12–24 weeks, and there are no studies of longer duration. In addition, the quality of the included studies was low to moderate. Therefore, the long-term effect needs to be established by longer, larger, multicentre, and higher-quality clinical studies.

## Conclusion

This meta-analysis demonstrates that exenatide, either alone or in combination with metformin, exhibits superior performance over metformin in managing PCOS, while maintaining a comparable side effect profile. Exenatide, either alone or in combination with metformin, appears to be a safe and effective treatment choice for patients diagnosed with PCOS. Further studies with larger sample sizes and longer durations are warranted to investigate the combined treatment approach when obesity is linked to PCOS.

### Supplementary Information

**Additional file 1.** PRISMA 2009 Checklist.

**Additional file 2:** **Table S1.** The search history on PubMed. **Table S2.** RCT Risk of Bias Assessment. **Fig S1.** Effects on HOMA-IR, FBG and FINS between exenatide and metformin. **Fig S2.** Effects on Weight, BMI, WC, WHR between exenatide and metformin. **Fig S3.** Sensitivity analysis of TT and FAI. **Fig S4.** subgroup analyses of FBG. **Fig S5.** Subgroup analyses of BMI. **Fig S6.** Putative mechanism of beneficial effects of exenatide.

**Additional file 3.** Table for excluded literature in meta-analysis.

## Data Availability

The paper contains all the evidence that supports the results. The corresponding author will provide more in-depth information and raw data upon reasonable request.

## Abbreviations

PCOS: Polycystic ovary syndrome
GLP-1: Glucagon-like peptide-1
RCTs: Randomized controlled trials
MFR: Menstrual frequency ratio
IR: Insulin resistance
TT: Total testosterone
FSH: Follicle-stimulating hormone
LH: Luteinizing hormone
DHEAS: Dehydroepiandrosterone sulfate
SHBG: Sex hormone-binding globulin
FAI: Free androgen index
HOMA-IR: Homeostasis model assessment-insulin resistance
FINS: Fasting insulin
FBG: Fasting blood glucose
BMI: Body mass index
WC: Waist circumference
WHR: Waist-hip ratio
GRADE: Grading of Recommendations and Evaluation
SMD: Standardized mean difference
MD: Mean difference
CI: Confidence interval
RR: Risk ratio
