# Supplementary material for: Comparison of exenatide alone or combined with metformin versus metformin in the treatment of polycystic ovaries: a systematic review and meta-analysis
Source: BMC Endocr Disord. 2023 Nov 16;23:250. doi: 10.1186/s12902-023-01497-x (PMC10652559; doi:10.1186/s12902-023-01497-x)
Supplement: Supplementary file 2 — Additional file 2: Table S1. The search history on PubMed. Table S2. RCT Risk of Bias Assessment. Fig S1. Effects on HOMA-IR, FBG and FINS between exenatide and metformin. Fig S2. Effects on Weight, BMI, WC, WHR between exenatide and metformin. Fig S3. Sensitivity analysis of TT and FAI. Fig S4. subgroup analyses of FBG. Fig S5. Subgroup analyses of BMI. Fig S6. Putative mechanism of beneficial effects of exenatide. [file 12902_2023_1497_MOESM2_ESM.docx]

Table S1. The search history on PubMed

| Search number | Query | Results |
| --- | --- | --- |
| #1 | "Polycystic Ovary Syndrome"[Mesh] | 18,149 |
| #2 | (((((((((((((Ovary Syndrome, Polycystic[Title/Abstract])) OR (Syndrome, Polycystic Ovary[Title/Abstract])) OR (Stein-Leventhal Syndrome[Title/Abstract])) OR (Stein Leventhal Syndrome[Title/Abstract])) OR (Syndrome, Stein-Leventhal[Title/Abstract])) OR (Sclerocystic Ovarian Degeneration[Title/Abstract])) OR (Ovarian Degeneration, Sclerocystic[Title/Abstract])) OR (Sclerocystic Ovary Syndrome[Title/Abstract])) OR (Polycystic Ovarian Syndrome[Title/Abstract])) OR (Ovarian Syndrome, Polycystic[Title/Abstract])) OR (Polycystic Ovary Syndrome 1[Title/Abstract])) OR (Sclerocystic Ovaries[Title/Abstract])) OR (Sclerocystic Ovary[Title/Abstract]) | 4,702 |
| #3 | ("Polycystic Ovary Syndrome"[Mesh]) OR ((((((((((((((Ovary Syndrome, Polycystic[Title/Abstract])) OR (Syndrome, Polycystic Ovary[Title/Abstract])) OR (Stein-Leventhal Syndrome[Title/Abstract])) OR (Stein Leventhal Syndrome[Title/Abstract])) OR (Syndrome, Stein-Leventhal[Title/Abstract])) OR (Sclerocystic Ovarian Degeneration[Title/Abstract])) OR (Ovarian Degeneration, Sclerocystic[Title/Abstract])) OR (Sclerocystic Ovary Syndrome[Title/Abstract])) OR (Polycystic Ovarian Syndrome[Title/Abstract])) OR (Ovarian Syndrome, Polycystic[Title/Abstract])) OR (Polycystic Ovary Syndrome 1[Title/Abstract])) OR (Sclerocystic Ovaries[Title/Abstract])) OR (Sclerocystic Ovary[Title/Abstract])) | 19,721 |
| #4 | "Exenatide"[Mesh] | 2,904 |
| #5 | (((((((((Bydureon[Title/Abstract])) OR (ITCA 650[Title/Abstract])) OR (AC 2993 LAR[Title/Abstract])) OR (Exendin-4[Title/Abstract])) OR (Ex4 Peptide[Title/Abstract])) OR (Peptide, Ex4[Title/Abstract])) OR (Exendin 4[Title/Abstract])) OR (Byetta[Title/Abstract])) OR (AC 2993[Title/Abstract]) | 2,079 |
| #6 | ("Exenatide"[Mesh]) OR ((((((((((Bydureon[Title/Abstract])) OR (ITCA 650[Title/Abstract])) OR (AC 2993 LAR[Title/Abstract])) OR (Exendin-4[Title/Abstract])) OR (Ex4 Peptide[Title/Abstract])) OR (Peptide, Ex4[Title/Abstract])) OR (Exendin 4[Title/Abstract])) OR (Byetta[Title/Abstract])) OR (AC 2993[Title/Abstract])) | 3,639 |
| #7 | (("Polycystic Ovary Syndrome"[Mesh]) OR ((((((((((((((Ovary Syndrome, Polycystic[Title/Abstract])) OR (Syndrome, Polycystic Ovary[Title/Abstract])) OR (Stein-Leventhal Syndrome[Title/Abstract])) OR (Stein Leventhal Syndrome[Title/Abstract])) OR (Syndrome, Stein-Leventhal[Title/Abstract])) OR (Sclerocystic Ovarian Degeneration[Title/Abstract])) OR (Ovarian Degeneration, Sclerocystic[Title/Abstract])) OR (Sclerocystic Ovary Syndrome[Title/Abstract])) OR (Polycystic Ovarian Syndrome[Title/Abstract])) OR (Ovarian Syndrome, Polycystic[Title/Abstract])) OR (Polycystic Ovary Syndrome 1[Title/Abstract])) OR (Sclerocystic Ovaries[Title/Abstract])) OR (Sclerocystic Ovary[Title/Abstract]))) AND (("Exenatide"[Mesh]) OR ((((((((((Bydureon[Title/Abstract])) OR (ITCA 650[Title/Abstract])) OR (AC 2993 LAR[Title/Abstract])) OR (Exendin-4[Title/Abstract])) OR (Ex4 Peptide[Title/Abstract])) OR (Peptide, Ex4[Title/Abstract])) OR (Exendin 4[Title/Abstract])) OR (Byetta[Title/Abstract])) OR (AC 2993[Title/Abstract]))) | 20 |
| #8 | (((randomized controlled trial [Publication Type])) OR (randomized [Title/Abstract])) OR (placebo [Title/Abstract]) | 1026,933 |
| #9 | ((((randomized controlled trial[Publication Type])) OR (randomized[Title/Abstract])) OR (placebo[Title/Abstract])) AND ((("Polycystic Ovary Syndrome"[Mesh]) OR ((((((((((((((Ovary Syndrome, Polycystic[Title/Abstract])) OR (Syndrome, Polycystic Ovary[Title/Abstract])) OR (Stein-Leventhal Syndrome[Title/Abstract])) OR (Stein Leventhal Syndrome[Title/Abstract])) OR (Syndrome, Stein-Leventhal[Title/Abstract])) OR (Sclerocystic Ovarian Degeneration[Title/Abstract])) OR (Ovarian Degeneration, Sclerocystic[Title/Abstract])) OR (Sclerocystic Ovary Syndrome[Title/Abstract])) OR (Polycystic Ovarian Syndrome[Title/Abstract])) OR (Ovarian Syndrome, Polycystic[Title/Abstract])) OR (Polycystic Ovary Syndrome 1[Title/Abstract])) OR (Sclerocystic Ovaries[Title/Abstract])) OR (Sclerocystic Ovary[Title/Abstract]))) AND (("Exenatide"[Mesh]) OR ((((((((((Bydureon[Title/Abstract])) OR (ITCA 650[Title/Abstract])) OR (AC 2993 LAR[Title/Abstract])) OR (Exendin-4[Title/Abstract])) OR (Ex4 Peptide[Title/Abstract])) OR (Peptide, Ex4[Title/Abstract])) OR (Exendin 4[Title/Abstract])) OR (Byetta[Title/Abstract])) OR (AC 2993[Title/Abstract])))) | 9 |

Table S2. RCT Risk of Bias Assessment

| Study | Random sequence generation | Allocation concealment | Blinding of participants and personnel | Blinding of outcome assessment | Incomplete outcome data | Optional report results | Other sources of bias |
| --- | --- | --- | --- | --- | --- | --- | --- |
| Elkind-Hirsch. K et al, 2008[31] | Patients were randomized through computer generated  Low Risk | Low Risk | No blinding  High Risk | NA  Unclear Risk | Drop-out rate (20%) Missing data with reasons given  Low Risk | A priori outcomes reported in main report Protocol available  Low Risk | No bias identified  Low Risk |
| Li. R.Y et at 2020[17] | Patients were randomized by a computer-generated system  Low Risk | Low Risk | NA  Unclear Risk | NA  Unclear Risk | NA  Unclear Risk | A priori outcomes reported in main report Protocol available  Low Risk | No bias identified  Low Risk |
| Li. R et al,2022[16] | Patients were randomized through a randomization list using statistics analysis system  Low Risk | Low Risk | No blinding  High Risk | NA  Unclear Risk | Low drop-out rate (8%) Missing data with reasons given  Low Risk | A priori outcomes reported in main report Protocol available  Low Risk | No bias identified  Low Risk |
| Liu. X et al,2017 [18] | Patients were randomized through computer generated  Low Risk | Low Risk | No blinding  High Risk | NA  Unclear Risk | Drop-out rate (10%) Missing data with reasons given  Low Risk | A priori outcomes reported in main report Protocol available  Low Risk | No bias identified  Low Risk |
| Ma. R. L et al, 2021[21] | The randomized codes were generated electronically using a two-block randomization technique to create a treatment allocation spreadsheet.  Low Risk | Low Risk | NA  Unclear Risk | NA  Unclear Risk | Drop-out rate (20%) Missing data with reasons given  Low Risk | A priori outcomes reported in main report Protocol available  Low Risk | No bias identified  Low Risk |
| Tao. T et al,2021 [32] | Patients were randomized by a computer-generated coding system.  Low Risk | Low Risk | No blinding High Risk | NA  Unclear Risk | Drop-out rate (18%) Missing data with reasons given  Low Risk | A priori outcomes reported in main report Protocol available  Low Risk | No bias identified  Low Risk |
| Wang. J et al, 2017[33] | Patients were randomized through random number table  Low Risk | Low Risk | Blinding  Low Risk | No blinding High Risk | No participants drop-out  Low Risk | A priori outcomes reported in main report Protocol available Low Risk | No bias identified  Low Risk |
| Zheng. S et al, 2017[10] | Patients were randomized by a computer-generated system  Low Risk | Low Risk | NA  Unclear Risk | NA  Unclear Risk | Drop-out rate (23%) Missing data with reasons given  Low Risk | A priori outcomes reported in main report Protocol available  Low Risk | No bias identified  Low Risk |
| Zheng. S et al, 2019[20] | Patients were randomized through randomizing software  Low Risk | Low Risk | NA  Unclear Risk | NA  Unclear Risk | Drop-out rate (23%) Missing data with reasons given  Low Risk | A priori outcomes reported in main report Protocol available  Low Risk | No bias identified  Low Risk |

NA, Not Available


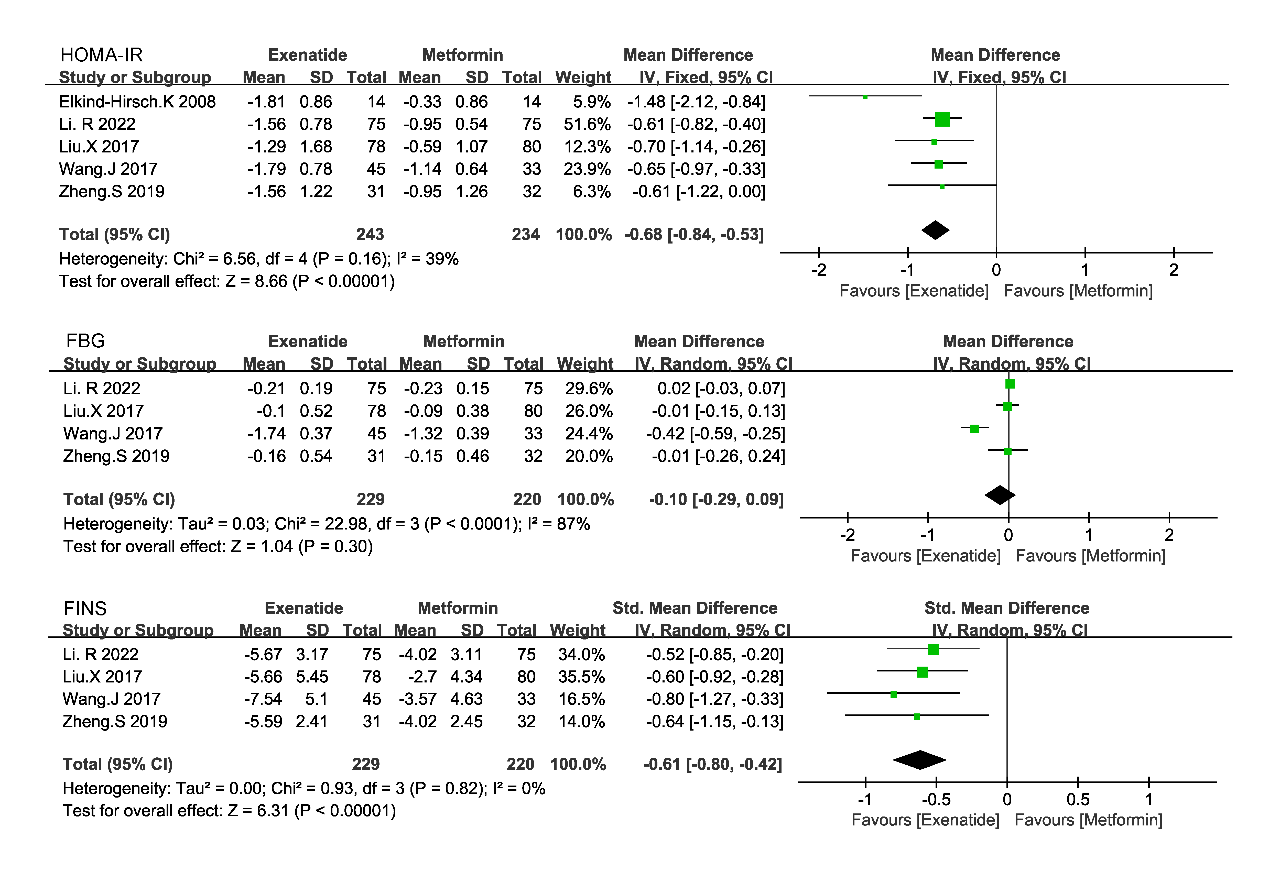


Fig S1 Effects on HOMA-IR, FBG and FINS between exenatide and metformin


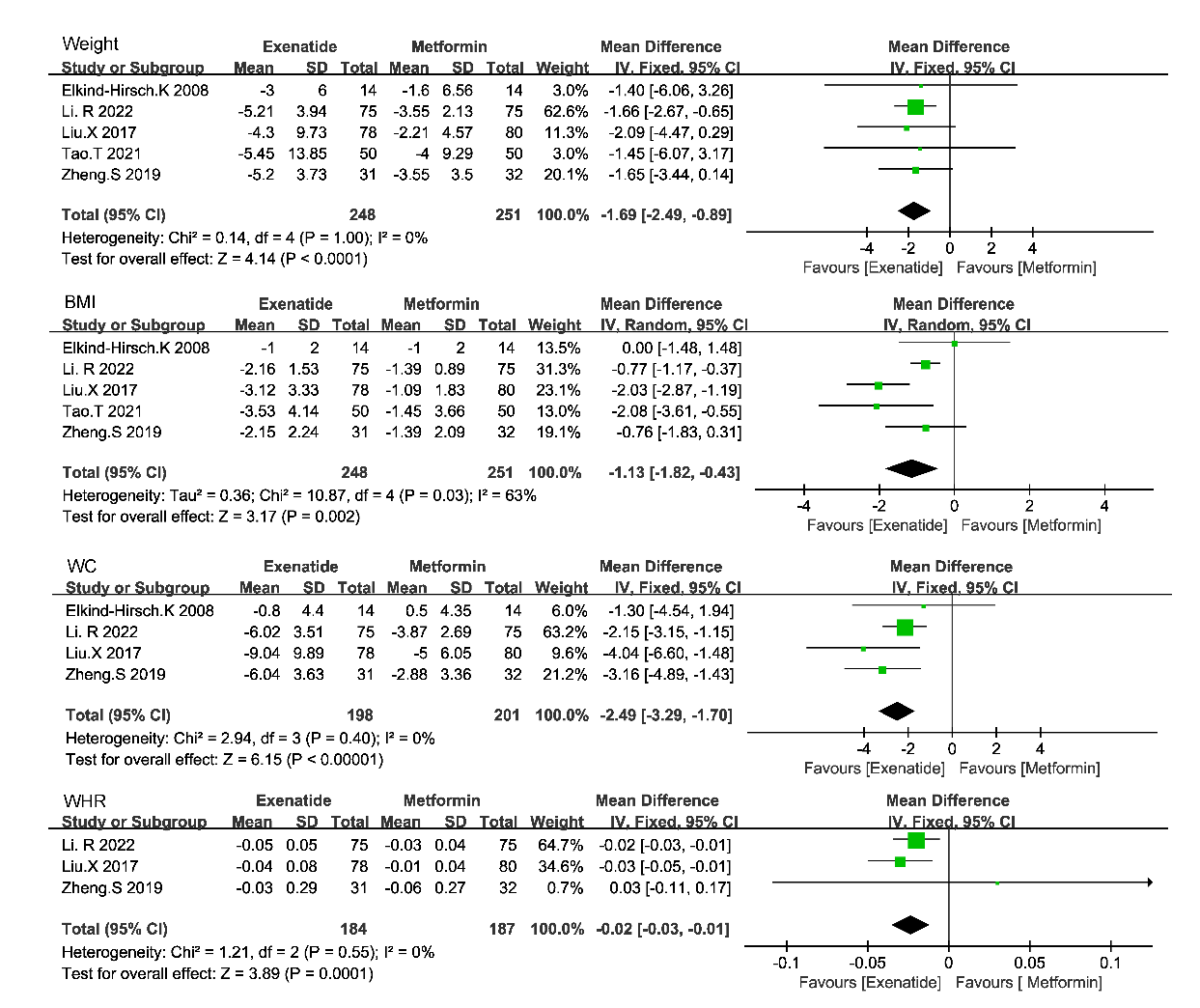


Fig S2 Effects on Weight, BMI, WC, WHR between exenatide and metformin


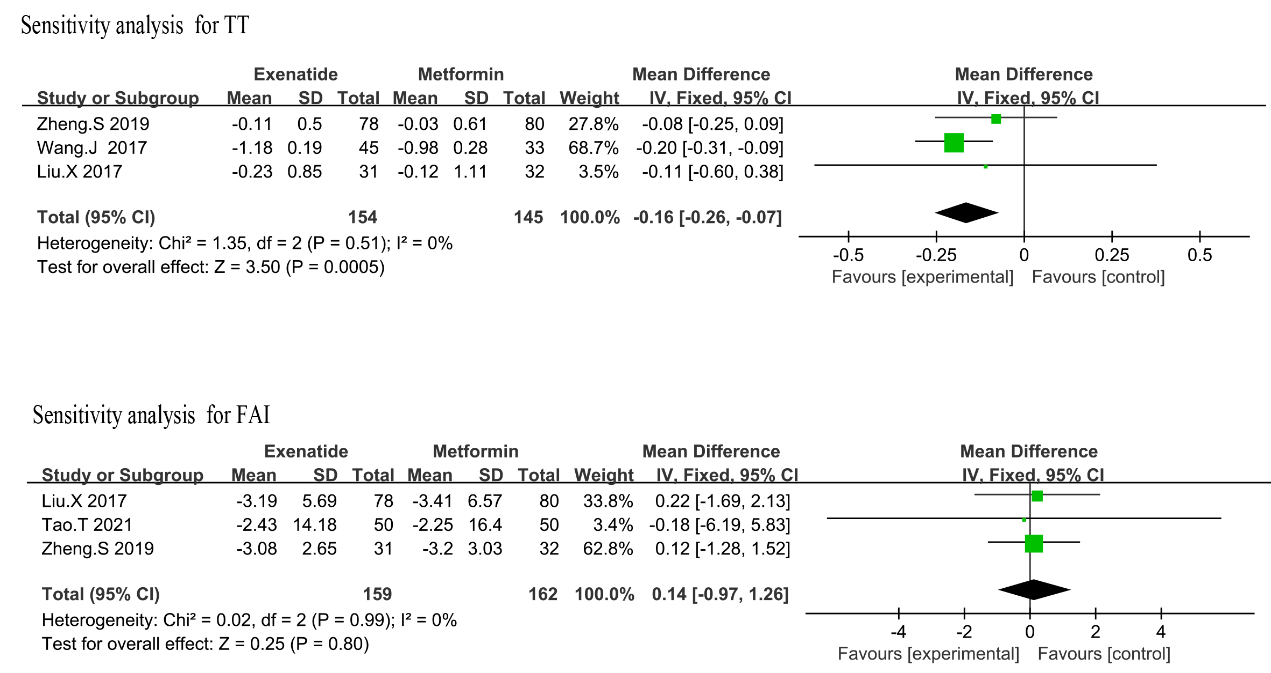


Fig S3 sensitivity analysis of TT and FAI


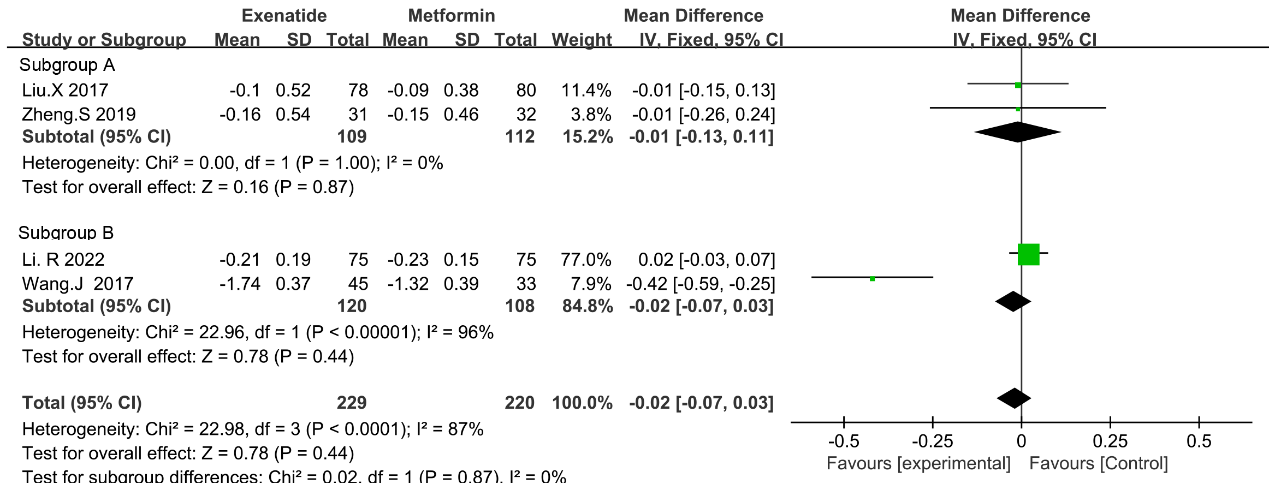


Fig S4 subgroup analyses of FBG

The difference in FBG between the two groups was insignificant. Due to the heterogeneity among the four studies included in the FBG meta-analysis, we conducted subgroup analyses. In the studies "Liu X 2017" and "Zheng. S 2019", the exenatide dosage was 10 μg bid, and the metformin dosage in the control group was 1000 mg bid. In contrast, in the "Li R 2022" study, the exenatide dosage was 5 μg bid and the metformin dosage in the control group was 1000 mg bid. In the "Wang J 2017" study, the exenatide dosage was 10 μg bid and the metformin dosage in the control group was 1000 mg tid. (Table 1) After conducting the subgroup analysis based on the dosages of exenatide and metformin, no further heterogeneity was observed in subgroup A (I²=0%, P=0.99). The results of the subgroup analysis were consistent with the previous findings.


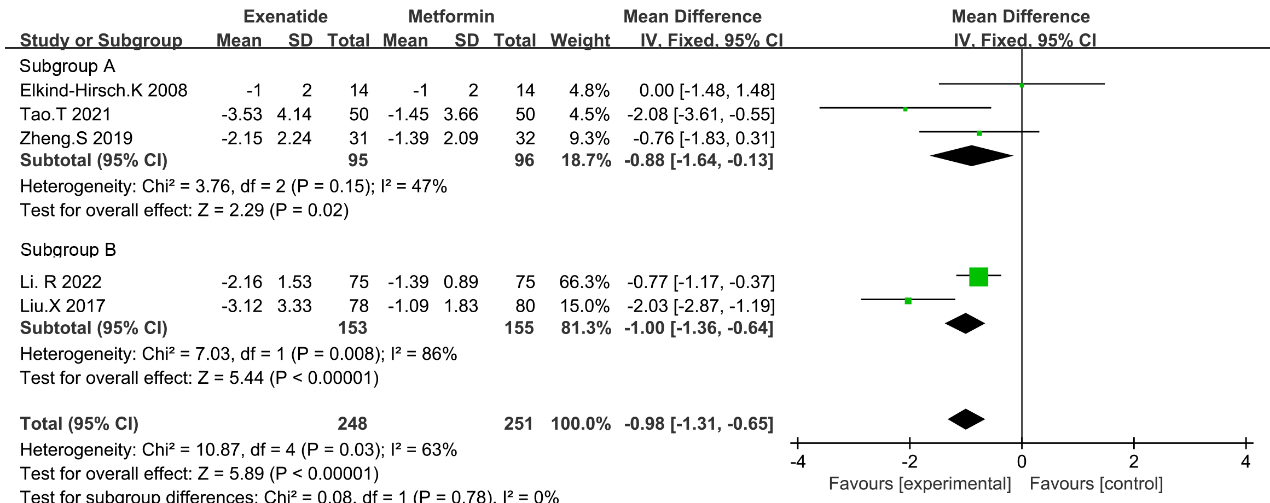


Fig S5 subgroup analyses of BMI

In 5 RCTs, BMI was reported for 449 patients. A random-effects model was utilized for analysis, and the results showed that exenatide resulted in a greater reduction in BMI than metformin. Due to the heterogeneity among the four studies included in the BMI meta-analysis, we conducted subgroup analyses. Furthermore, due to the heterogeneity among the five studies included in the BMI meta-analysis, we conducted a more specific subgroup analysis. According to three studies, "Elkind-Hirsch K 2008", "Tao T 2021", and "Zheng. S 2019", patients in the exenatide group had a higher initial body weight than those in the metformin control group. On the other hand, the studies "Li R 2022" and "Liu X 2017" reported that patients in the exenatide group had a lower initial body weight than those in the metformin group. After performing the subgroup analysis, we found that the heterogeneity in subgroup A was minimal (I²=47%, P=0.15). The results of this subgroup analysis were consistent with our initial findings.

**
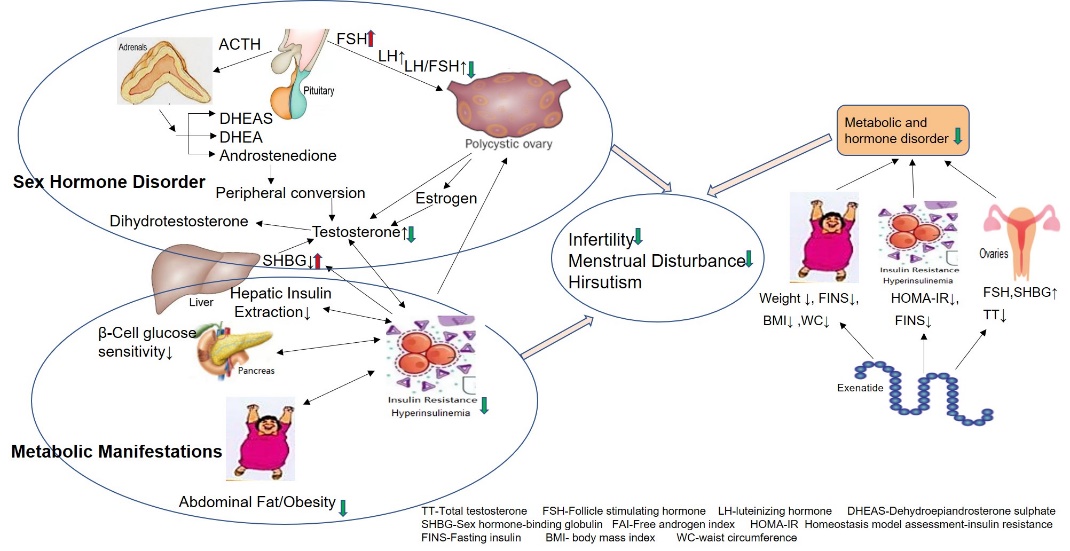
**

Fig S6 Putative mechanism of beneficial effects of exenatide
